# Retinal Pigment Epithelial Cell Line Suppression of Phagolysosome Activation

**Published:** 2015-01-29

**Authors:** AW Taylor, S Dixit, J Yu

**Affiliations:** Department of Ophthalmology, Boston University School of Medicine, Boston, MA, USA

**Keywords:** Alpha-Melanocyte Stimulating Hormone, Immune Privilege, Macrophages, Neuroimmunomodulation, Neuropeptide Y, Phagocytosis

## Abstract

The eye is an immune privileged tissue with multiple mechanisms of immunosuppression to protect the light gathering tissues from the damage of inflammation. One of theses mechanisms involves retinal pigment epithelial cell suppression of phagosome activation in macrophages. The objective of this work is to determine if the human RPE cell line ARPE-19 is capable of suppressing the activation of the phagolysosome in macrophages in a manner similar to primary RPE. The conditioned media of RPE eyecups, sub-confluent, just confluent cultures, or established confluent cultures of human ARPE-19 cells were generated. These condition media were used to treat macrophages phagocytizing pHrodo bioparticles. After 24 hours incubation the macrophages were imaged by fluorescent microscopy, and fluorescence was measured. The fluorescent intensity is proportional to the amount of bioparticles phagocytized and are in an activated phagolysosome. The conditioned media of in situ mouse RPE eyecups significantly suppressed the activation of phagolysosome. The conditioned media from cultures of human ARPE-19 cells, grown to sub-confluence (50%) or grown to confluence had no effect on phagolysosome activation. In contrast, the conditioned media from established confluent cultures significantly suppressed phagolysosome activation. The neuropeptides alpha-MSH and NPY were depleted from the conditioned media of established confluent ARPE-19 cell cultures. This depleted conditioned media had diminished suppression of phagolysosome activation while promoting macrophage cell death. In addition, the condition media from cultures of ARPE-19 monolayers wounded with a bisecting scrape was diminished in suppressing phagolysosome activation. This technical report suggests that like primary RPE monolayers, established confluent cultures of ARPE-19 cells produce soluble factors that suppress the activation of macrophages, and can be used to study the molecular mechanisms of retinal immunobiology. In addition, the results further demonstrate the importance of an intact monolayer of RPE cells to modulate immune cell activity within the eye.

## Introduction

The ocular microenvironment is immunosuppressive. This immunosuppression is seen as the inhibition of inflammatory activity within the eye. This involves regulating the resident, and infiltrating immune cells within the ocular tissues. One of the immunosuppressive mechanisms is mediated by a specific set of soluble neuropeptides, and growth factors found in aqueous humor, and produced by retinal pigment epithelial cells (RPE) [1–5]. Macrophages stimulated to mediate inflammation are suppressed when treated with aqueous humor, or to the soluble factors made by a healthy RPE monolayer. Moreover, the macrophages are induced to mediate anti-inflammatory activity, and promote the activation of regulatory T cells. It is considered that this is part of the endogenous mechanisms of ocular immune privilege to protect the delicate structure, and light gathering functions of the eye from the collateral damage of inflammation, and autoimmune disease.

Two of the soluble neuropeptides made by the RPE are alphamelanocyte stimulating hormone (α-MSH) and neuropeptide Y (NPY) [4]. These two neuropeptides are responsible for RPE inducing characteristics of myeloid suppressor cells in macrophages, and promoting the same characteristics in retinal microglial cells [4]. A part of this suppression is the α-MSH and NPY modulating phagocytic activity in macrophages [6]. Macrophages treated with α-MSH and NPY are able to take up bacterial-bioparticles, but are suppressed in moving the bioparticles to an active phagolysosome. This may be part of an ocular immunosuppressive mechanism to minimize the chance of presenting processed autoantigens, while allow for clearance of dead cells and metabolic byproducts.

The finding of RPE immunomodulation has involved isolating and culturing the primary RPE cells that proliferate into a monolayer [5,7]. This is a process that is not usually seen except in wounded retinas. Also, RPE immunomodulation has been studied by making of posterior eyecups that don’t disrupt the RPE monolayer, but are short lived cultures [2,3]. In both, most of the studies are with mouse eye cells, because they are more easily obtained, and that different intraocular conditions can be manipulated prior to collecting the RPE. While it is assumed that the RPE from a healthy cadaver eye will suggest homeostatic immunoregulating activity, it is difficult to make any initial studies into the effects of different ocular microenvironments from RPE in humans. One way around this limitation is to use the human RPE cell line ARPE-19. These cells grow to a confluent monolayer, and have been used to study the effects of oxidants and cytokines involved in RPE changes related to age-related macular degeneration [8,9]. These cell lines when treated with TGF-β2 have been found to mediate the activation of Treg cells [10]. The activity in these studies have shown parallel effects as seen in the eye with AMD, or by primary RPE cell cultures. Therefore, this cell line, under the right tissue culture conditions, can closely mimic the primary RPE. They make it possible to characterize immune homeostatic activity of RPE, and to see what happens when the RPE is exposed to inflammatory mediators, or bacterial products. Since we have previously demonstrated that the conditioned media of our in situ RPE eyecup cultures modulate the activation of the phagolysosome [6], in this report we show that ARPE-19 cells similarly suppress the activation of the phagolysosomes in macrophages that have phagocytized bioparticles.

## Materials and Methods

### RPE Eyecups

Eyecups with intact RPE monolayers were prepared as previously described [1,3,4]. In brief, eyes from euthanized healthy C57BL/6J mice were enucleated, placed in ice cold DMEM (Lonza, Walkersvile, PA), where external muscles, and connective tissue were cut from the eyes. A circumferential incision was performed below the level of the ciliary body, and the anterior segment and lens was discarded. The neural retina was gently lifted off the RPE by microsurgical forceps. Each RPE eyecup were placed into individual wells of a 96-well round-bottom culture plate submerged in 200 μL of serum-free media consisting of RPMI 1640, 0.1 M HEPES, NEAA, L- glutamine, sodium pyruvate, 0.1% bovine serum albumin, 1% gentamicin, and supplemented with 0.1 x ITS+ solution (Sigma Chemical, St. Louis, MO). The conditioned media (CM) from the eyecup cultures were removed 48 hours after incubation, and used in the assays.

### ARPE-19 Cell Cultures

The human retinal pigment epithelial cell line ARPE-19 (ATCC CRL-2302) was maintained in DMEM/F12 media (Lonza) plus 10% Fetal Bovine Serum (FBS) (Gibco-Life Technologies, Grand Island, NY ). The wells of a 24-well cell culture plate (Corning, Corning, NY) were seed with 1 x 10^5^ ARPE-19 cells, and incubated for 48 hours to grown to a confluent monolayer. To make established confluent monolayers, the media was changed, and the cells incubated for an additional 72 hours. For subconfluent cultures the wells were seeded with 2.4 x 10^4^ cells. In the last 24 hours of incubation the media was replaced with the serum-free media [6], and was used as conditioned media for the phagolysosome assays.

### Depletion of α-MSH and NPY

Antibodies to α-MSH and NPY (Bachem Americas Inc., Torrance, CA) were added to the ARPE-19 established monolayer conditioned media. For a control ChromPure Rabbit IgG (Jackson ImmunoResearch Laboratory, West Grove, PA) was used. Antibody concentrations were 2 μg/ml, a level that would bind a molar equivalent of 1 mg of α-MSH and NPY. This far in excess of what has been measured in culture for RPE eyecups [4]. The conditioned media with antibody was incubated 1 hour at 37°C. Protein-A coated beads (Santa Cruz Biotechnology) were add and the mixture was incubated for an additional 1 hour at 37°C. The beads were centrifuged down, and the α-MSH/NPY-depleted supernatant was used in the phagolysosome assay.

### Macrophage Cultures and Phagolysosome Assay

The BALB/c-derived mouse macrophages, RAW 264.7 (ATCC TIB-71), were seeded at 1 x 10^5^ cells into 35mm tissue culture dishes. The cells were incubated for 1 hour, and into the wells was added the conditioned media of incubated RPE eyecups, or ARPE-19 cultures at no more than a 1:2 dilution. After an additional 30 min of incubation, the pHrodo-Staphylococcus aureus bioparticles (0.02 mg) were added to the cultures. The cultures were incubated for 24 hours and the cells were imaged for fluorescence.

### Imaging

The cultures were digitally imaged with an Olympus FSX100 fluorescent microscope at a constant time of exposure. For each tissue culture dish, five images were captured. The relative corrected total cell fluorescence (CTCF) minus the background was calculated. The results are presented as fluorescence (mean ± SD) relative to the mean auto-fluorescence background of macrophages with no beads (resting cells).

### Statistical Analysis

Nonparametric Mann-Whitney U test were run on the results to assay for statistical significance between the average values for each condition with the positive and negative controls. All experiments are the results of 3 to 4 cultures run at different times. Statistical difference was detected with P ≤ 0.05.

## Results

### The Effects of RPE Eyecup Conditioned Media on Macrophage Phagolysosome Activation

**Figure 1:**
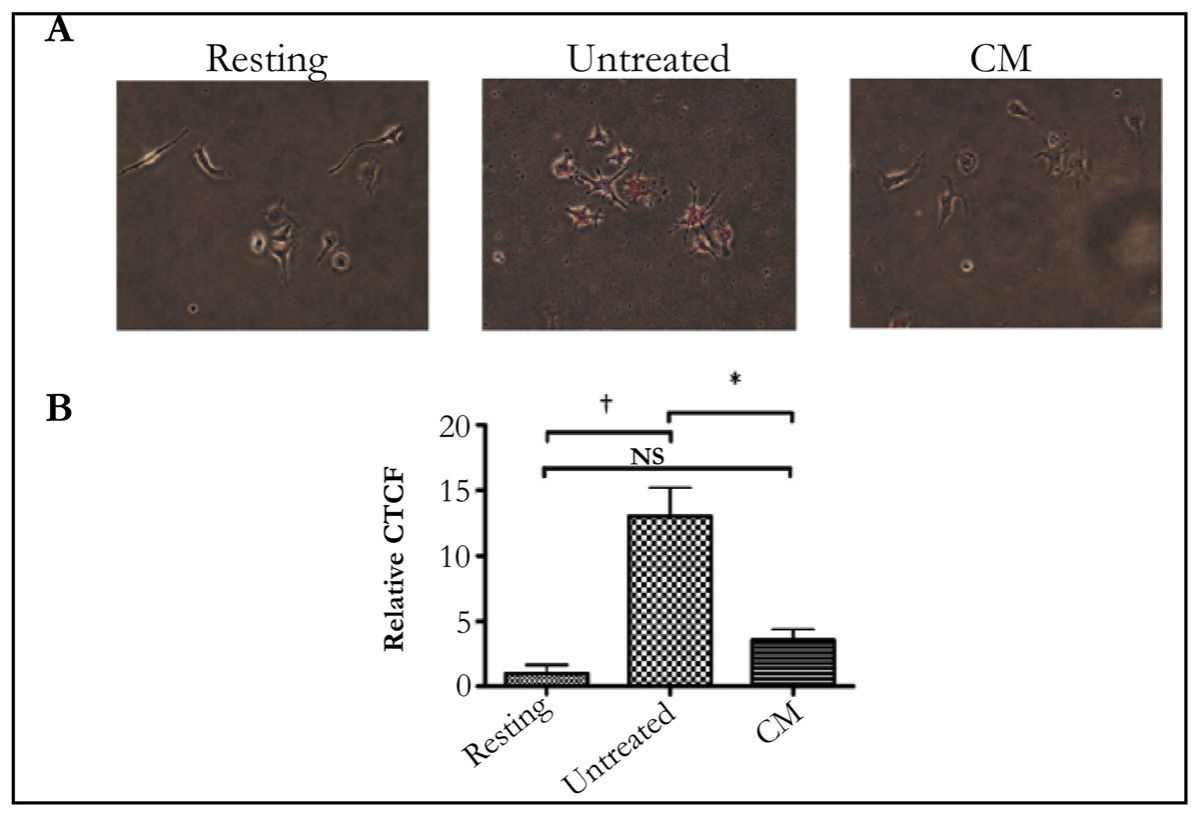
The conditioned media of RPE eyecups suppresses pHrodo-bioparticle fluorescence in macrophages. The macrophages were cultured as described, and treated with the conditioned media of RPE eye cups (CM) for 24 hours A) Representative images of the florescence observed with resting, untreated, and CM-treated macrophages. B) The corrected total cellular fluorescence (CTCF) was calculated and made relative to macrophages that were not given beads (Resting). The results are the mean ± SD of 4 cultures analyzing a total of 80 – 100 cells per condition. *Significantly different P ≤ 0.01, and NS is not significant. †P≤ 0.001. The RPE conditioned media suppressed the fluorescence of pHrodo-bioparticles in macrophages indicating suppression of the activation of the phagolysosome.

Previously it was demonstrated that the RPE neuropeptides α-MSH and NPY suppress the activation of the phagolysosome [6]. This was assayed by feeding macrophages, RAW 264.7 cells, with pHrodo-bioparticles that fluoresce red under acidic conditions (Fig. 1A). To see if primary RPE cell monolayers express a soluble factor that suppresses the activation of the phagolysosome in situ eye cups from healthy mouse eyes were cultured for 24 hours and the conditioned media was used to treat the macrophages before given pHrodo-bioparticles. After an additional 24 hours of incubation the cells were assayed for fluorescence. There was a significant suppression in fluorescence of macrophages treated with the eyecup conditioned media (Fig. 1B). Therefore, soluble immunomodulators produced by healthy RPE suppress the activation of the phagolysosome in macrophages.

### The Effects of Cultured Arpe-19 Cells on Macrophage Phagolysosome Activation

**Figure 2:**
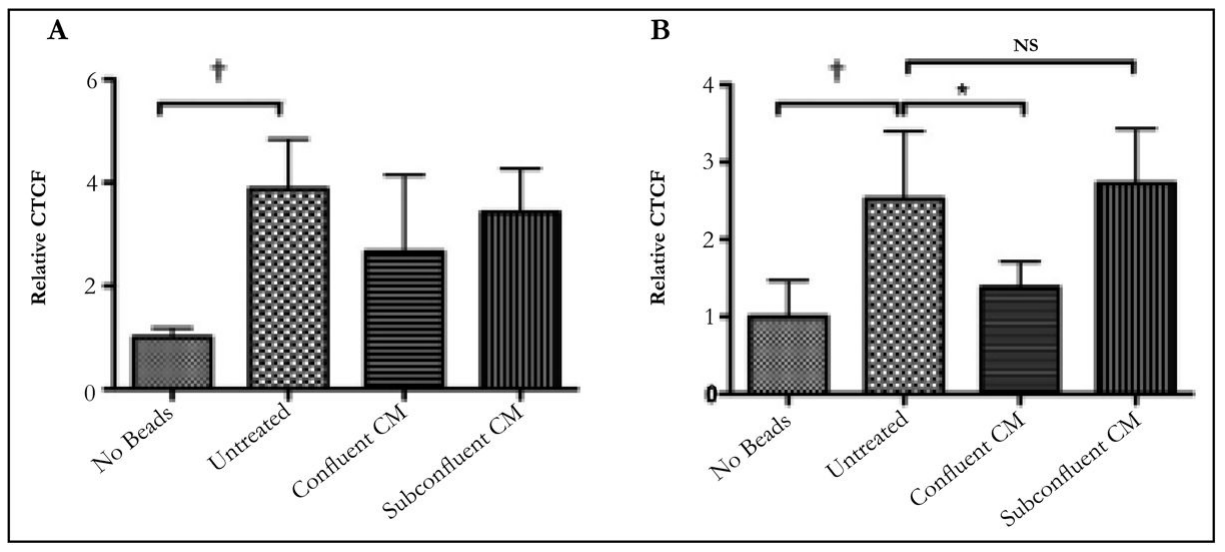
The conditioned media from established ARPE-19 confluent cultures suppressed pHrodo-bioparticle fluorescence in macrophages A) The ARPE-19 cells were grown to confluence, or kept subconfluent before changing the media for serum-free media. After 24 hours incubation in serum-free media the conditioned media (CM) was collected, and the macrophages were treated with the CM before fed pHrodo-bioparticles. 24 hours later the cells were assayed for fluorescence. No significant difference was seen between the pHrodo-bioparticle fluorescence in macrophages treated with CM from confluent or subconfluent cultures with untreated macrophages. B)The confluent ARPE-19 cultures were incubated for an additional 2 days before the media was changed to serum-free. *Significant (P≤0.01) differences were seen between the established confluent CM treated macrophages and the untreated macrophages. NS is not significant. †P ≤ 0.01. The results are the mean ± SD of 4 cultures analyzing a total of 50 – 75 cells per condition. Only the established confluence cultures of ARPE-19 cell produced soluble factors that suppressed the activation of the phagolysosome.

Since there are many reports showing almost the same activity of ARPE-19 cell monolayers to cultured primary RPE [8–11], the conditioned media of cultured ARPE-19 cells were assayed to see whether they suppress phagolysosome activation in phagocytizing macrophages. The ARPE-19 cells were cultured to confluence, or kept subconfluent before changing the media to the serum-free media for an additional 24 hours of incubation. The macrophages were treated with the conditioned media before given pHrodobioparticles to phagocytize. The cells were assayed 24 hours later for fluorescence. There was no significant change in the fluorescence between the macrophages treated with conditioned media from confluent or sub confluent ARPE-19 cultures to the untreated macrophages (Fig. 2A).

To generate conditioned media from established confluent monolayers, the ARPE-19 cells were grown to confluence and maintained at confluence for 3 days before changing the media for serum- free. The conditioned media from these cultures significantly suppressed phagolysosome activation in the phagocytizing macrophages compared to untreated macrophages, and macrophages treated with conditioned media from subconfluent ARPE-19 cell cultures (Fig. 2B). Therefore, ARPE-19 cells produce soluble immunomodulators when they are an established confluent culture.

### The role of α-MSH and NPY in ARPE-19 suppression of phagolysosome activation

**Figure 3:**
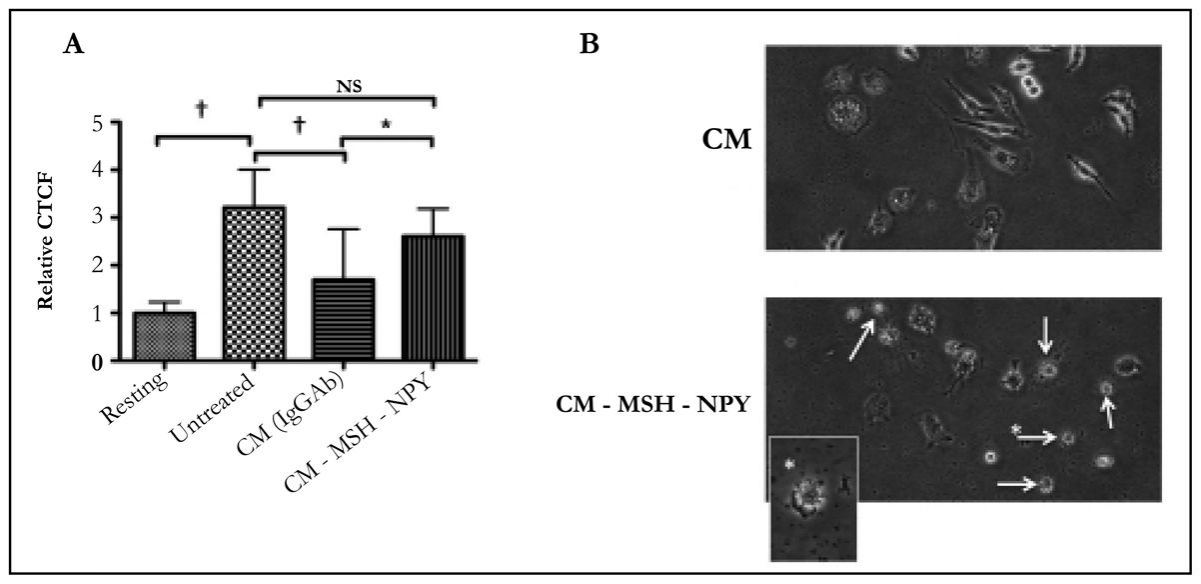
The depletion of α-MSH and NPY from CM of established confluent APRE-19 cultures removes the suppression of pHrodo-bioparticle fluorescence in macrophages A) The conditioned media of established ARPE-19 cell cultures antibody depleted of α-MSH and NPY were used to treat the macrophages that were then fed pHrodo-bioparticles. *There was a significant (P ≤ 0.01) increase in the pHrodo-bioparticles fluorescence in macrophages treated with CM depleted of α-MSH and NPY (CM-MSH-NPY) with macrophages treated with CM absorbed with irrelevant antibody (CM(IgGAb)). This was not significantly different from the untreated macrophages. Presented are the mean ± SD of 4 cultures analyzing a total of 50 – 75 cells per condition. B) The images of macrophages treated with CM(IgGAb) was compared with the images of the macrophages treated with the α-MSH/NPY-depleted CM. Arrows indicate dead or possible apoptotic cells. The arrow with the star marks the cell that was magnified in the figure inset. The ARPE-19 suppression of phagolysosome activation is mediated by at least α-MSH and NPY, and their depletion from the conditioned media causes macrophage cell death similar to what has been reported for conditioned media of RPE eyecups [4].

Since the neuropeptides α-MSH and NPY have an important role in RPE regulation of macrophage activity, the α-MSH and NPY were depleted in the conditioned media of established confluent ARPE-19 cell cultures by absorption with specific antibodies. The α-MSH/NPY-depleted conditioned media was significantly diminished in suppressing the activation of phagolysosomes in the treated macrophages (Fig. 3A). However, this diminishment is possibly due to less viable cells remaining in culture after treatment with α-MSH/NPY-depleted conditioned media. Morphologically the macrophages treated with the depleted conditioned media were rounded with numerous vacuoles with the appearance of cells undergoing apoptosis (Fig. 3B). This is in comparison to the abundance of large, dendritic, and intact healthy macrophages treated with whole conditioned media (Fig. 3B). A similar morphological effect was reported when α-MSH and NPY were depleted from the conditioned media of healthy RPE eyecups [3]. Therefore, the modulation of macrophage functionality and survival by established confluent cultures of ARPE-19 cells, like primary RPE, includes α-MSH and NPY.

### The effects of wounding the ARPE-19 established confluent cultures

**Figure 4:**
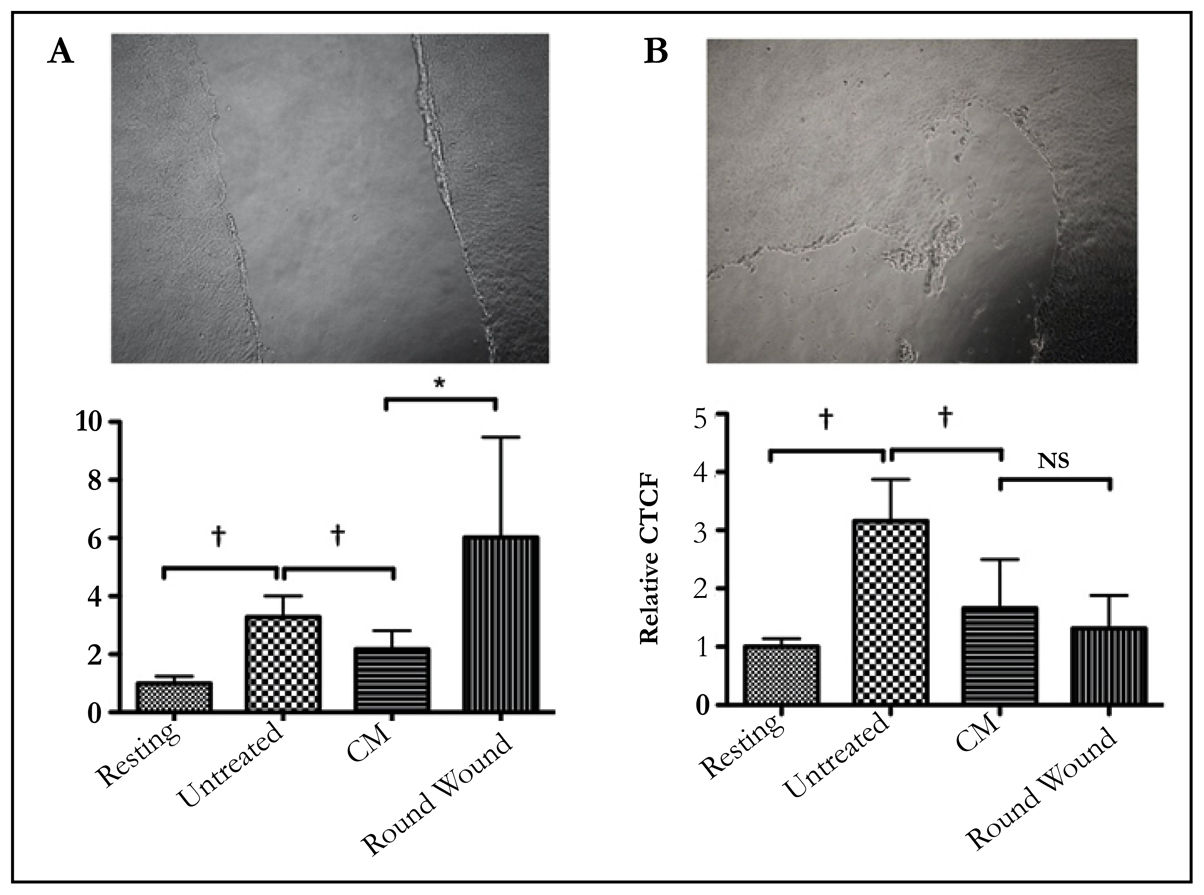
The effects of disrupting the ARPE-19 established confluent monolayer. Established ARPE-19 confluent monolayers were A) bisected with a scrape, or B) wounded with round scrapes before the cell were cultured in serum-free media. Presented are representative phase contract micrographs of the wounded cultures and the relative effects of the conditioned media on phagolysosome activation. *Significant (P ≤ 0.05) loss of suppression was seen with the conditioned media from ARPE-19 cultures with a bisecting scrape wound, but not with rounded wounds. Moreover the pHrodo-bioparticles fluorescence in macrophages treated with CM from the bisected scrape cultures had enhanced levels of fluorescence. †P ≤ 0.05. The results are the mean ± SD of 4 cultures analyzing a total of 50 – 75 cells per condition. An intact confluent monolayer necessary for ARPE-19 cells to suppress the activation of the phagolysosome.

To see if maintaining the ARPE-19 confluent monolayer is required for ARPE-19 suppression of phagolysosome activation, the confluent cultures were subjected to either a scrape wound that bisected the monolayer, or to round wounds similar to laser wounding of the RPE monolayer in vivo. Conditioned media from these cultures were used to treat the macrophages before given pHrodo-bioparticles. When the conditioned media was from a scrape wound that broke the confluent culture into separate parts there was a significant loss in suppressing the activation of the phagolysosome (Fig. 4A). Moreover, many of the macrophages were enhanced in their phagocytic activity although not significantly different from untreated macrophages. In contrast, the conditioned media from the ARPE-19 cultures with round wounds continued to significantly suppress phagolysosome activation that was not significantly different from conditioned media of intact ARPE-19 confluent cultures (Fig. 4B). Therefore, wounding the established ARPE-19 confluent cultures in a manner that breaks the continuous connection of cells across the culture causes a loss of modulation activity by the cultured cells. This suggests that the production of immunomodulating activity by the ARPE-19 cells is a result of connections that must form across the established confluent monolayer cells.

## Discussion

The RPE monolayer is expected to perform multiple activities to maintain the health and function of the retina [12]. It consumes the outer segments, and recycles and supplies nutrients for the photoreceptors. It is a physical barrier defining the ocular microenvironment. It is a source and mediator of immunomodulation necessary to prevent the activation of inflammation within the ocular microenvironment. The RPE suppress immunity by both cell surface and soluble molecules. On the surface of RPE are FasL, PD-L1, CTLA2, and TGF-β2 that with contact suppress effector T cell while inducing Treg cell activity [13–17]. In addition, soluble factors TGF-β2, Thrombospondin-1, and α-MSH further promote Treg cell activation [1,4]. It is the soluble factors produced by healthy RPE that affect the functionality of macrophages and microglial cells in the retina.

The neuropeptides α-MSH and NPY produced by the RPE are part of group of immunomodulating factors made by the RPE to suppress inflammatory activity by stimulated macrophages [4]. Theses two neuropeptides are responsible for RPE mediated induction of suppressor macrophages. In addition, their production by RPE promotes the expression of similar suppressor cell characteristics in retinal microglial cells [4]. To characterize the effects of RPE soluble factors on immune cells, the RPE were incubated as in situ posterior eyecups, and the conditioned media was collected and assayed. In a similar manner, the ARPE-19 conditioned media was collected and assayed. Supporting the original reason why the eyecups were used to keep the RPE monolayer, the ARPE-19 cells grown to an established monolayer were able to suppress the activation of the phagolysosome in the phagocytizing macrophages. Other studies have grown the ARPE-19 cells to form an established monolayer before being assayed [8]. It demonstrates that as an intact monolayer the ARPE-19 cell line is very useful in understanding the role of human RPE produced factors in modulating immunity.

When the RPE monolayer is wounded in mouse eyes, they are no longer able to promote the expression of myeloid-suppressor cell characteristics in macrophages, and in vivo similar characteristics in retinal microglial cells [4]. This has been associated with a change in RPE production of the neuropeptides. We were able to demonstrate that when the ARPE-19 monolayer was wounded with a bisecting scrape there was loss of RPE suppression of phagolysosome activation in the macrophages. In contrast, simple round wounds of the monolayer had no effect on the suppressive activity. This suggests that there is a limit to the amount of damage the RPE monolayer can sustain before it changes its functionality [11,18,19]. Also, damaged cells may release signals like damage associated molecular patterns (DAMPs) that promote macrophage activation. Therefore, wounding the ARPE-19 monolayer has similarities to the breakdown of the RPE monolayer in disease.

The conditioned media of RPE eyecups depleted of α-MSH and NPY promote the activation of M1-like macrophages; however, the lack of α-MSH causes the macrophages to die [4]. This has suggested that the RPE produce a pro-apoptotic signal in macrophages that is countered by α-MSH. The apoptotic signal blocked by α-MSH, or how α-MSH blocks apoptosis is not yet understood [20]. In parallel, the ARPE-19 conditioned media depleted of α-MSH and NPY were significantly diminished in their capacity to suppress activation of the phagolysosome in macrophages, and the macrophages treated with the depleted conditioned media are appearing to be dying. This demonstrates that the ARPE-19 monolayer, like the RPE eyecups, modify the phagolysosome activity in macrophages, and produce a death signal in the macrophages that is countered by the neuropeptides.

Having demonstrated that the confluent cultures of ARPE-19 cells are acting in a similar manner as the RPE eyecups, and that it is a similar manner between human and mouse RPE (both α-MSH and NPY are evolutionarily conserved), it should be possible to use these APRE-19 cultures to further clarify the soluble factors that are immunomodulating and pro-apoptotic on macrophages. Moreover, it should be possible to use the cultures of treated ARPE-19 cells to assay the effects of oxidative damage, bacterial products and other immune cell cytokines on RPE modulation of macrophage and microglial cell functionality.

The results further demonstrate that part of ocular immune privilege is RPE modulation of macrophage/microglial cell function in the retina. A healthy intact RPE monolayer is required for the maintenance of immune privilege. The combined effects of RPE soluble factors allows for macrophage phagocytosis, but prevents the conventional lysosomal degradation of the phagocytized materials, and the activation of inflammatory activity by the macrophages. This means that myeloid cells in the healthy eye are suppressed in phagocytic processing of antigens, and induction of inflammation, but not in phagocytic clearance and production of anti-inflammatory cytokines.
